# The application of orthodontic bone stretching for correcting malpositioned dental implants

**DOI:** 10.1186/s13005-021-00294-y

**Published:** 2021-10-14

**Authors:** Philippe Bousquet, Stéphane Barthélemi, Christèle Artz, Laurent Delsol

**Affiliations:** 1 Department of Periodontology, Dental Faculty, University of Montpellier, 545 Avenue Prof. JL Viala 34193 Cedex 5 Dr Philippe Bousquet, Montpellier, France; 2 Department of Orthodontics, Dental Faculty, University of Montpellier, 545 Avenue Pr JL Viala, 34193 Cedex 5 Montpellier, France

## Abstract

**Background:**

Dental implants are sometimes initially placed in a wrong position leading to esthetic damage, which is difficult to solve with prosthetics. Moreover, implants placed in the anterior sector, like ankylosed teeth, are frequently found in a wrong position over time with infraocclusion because of continuous anterior alveolar growth. Different treatments have been proposed to manage the consequences of malpositioned dental implants.

**Case presentation:**

This paper describes a surgical and orthodontic new procedure that can be used to relocate an implant in a wrong position: the Orthodontic Bone Stretching technique (OBS), which involves deep partial osteotomies combined with heavy orthodontic forces. The applied force facilitates esthetic rehabilitation with a movement towards the occlusal plane and can modify the implant axis and the gingival line alignment. This relocation is made possible thanks to a bone stretching phenomenon in the surgical area without immediate mobilization or repositioning of an alveolar segment. Three cases with the need for implant repositioning are presented here and were treated with the OBS technique.

**Conclusion:**

In the three cases presented, implant relocation was successfully performed with the OBS technique and the prosthetic crown was modified to improve esthetic results.

## Introduction

**Fig. 1:**
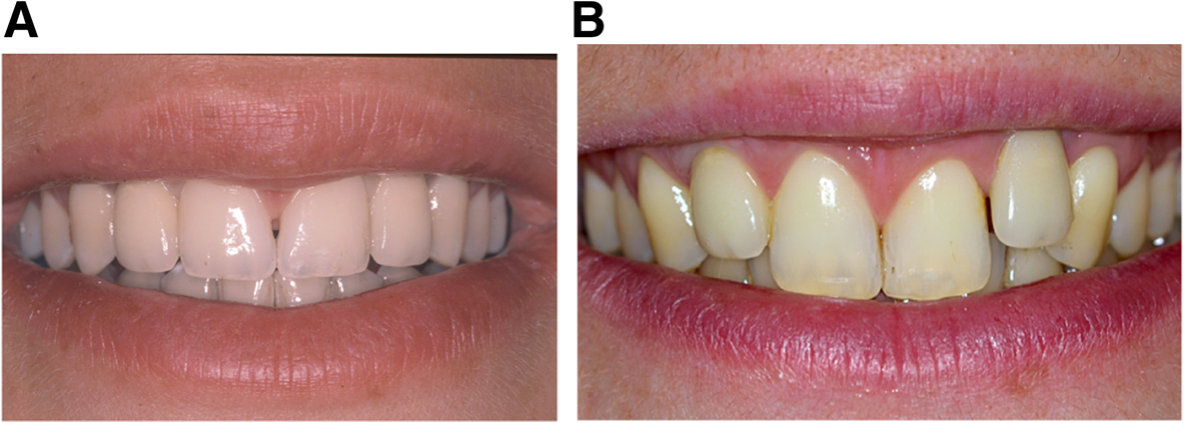
Missing lateral incisors case replaced by implants over the time. Missing lateral incisors case replaced by implants over the time. **A**: situation in 1998 at the end of implant treatment (Implantologist Dr. Philippe Russe). **B**: situation in 2014, revealing the severe misalignment of the implants 16 years later

Implant positioning in the anterior sector is always a challenge, as an ideal esthetic result is difficult to reach and maintain in the long term. Indeed, anterior alveolar growth, despite its decrease after adolescence, continues all life long and animal and clinical studies ([1–7]) have shown that implants do not follow the growth. This immobility can lead to infraocclusion and esthetic problems, particularly in young adults but also in older patients [6], with an increased risk for patients with an openbite facial pattern, and for women [8]. The lack of growth in the area surrounding the implant leads in many cases to a misalignment of adjacent teeth; this consequence has also been described for ankylosed teeth [8, 9]. (Fig. 1).

The improvement of implantology techniques, thanks to CFAO preparation and surgical guides, leads in most cases to an adequate implant positioning, but when the implant is initially malpositioned, esthetic failures will occur [10]. When an implant is placed too buccally with an inadequate angulation, the prosthetic result will lead to a crown which is too long with an incorrect alignment of the gingival line. This esthetic failure may increase over time in association with gingival recession [11].

When incorrect positioning is moderate, an issue is to use prosthetic individualized framework abutment as treatment. However, with important malpositions, prosthetic technique adaptation cannot lead to acceptable esthetic results. In these situations, it may be necessary to leave the implant submerged under the soft tissues or remove it surgically [12]. Implant removal frequently results in defects, requiring soft and hard tissue reconstruction as well as new implant insertion in a correct position. This procedure can be considered long and complex but has so far represented the best management for malpositioned implant associated with severe gingival recession or bone loss, with a poor prognosis from the esthetic point of view.

Ankylosed teeth may appear to be a good model of comparison with osseointegrated implants because of their behavior during growth or aging. Different surgical relocation procedures have been proposed in the literature to treat the consequences of tooth ankylosis as an alternative treatment for avulsion [13]. On the same model, if the implant is completely osseointegrated without hard and soft tissue defect but with an initial wrong implant positioning, different treatment options are proposed for implant relocation to provide an alternative to implant removal.

The first possibility is a segmental osteotomy with bony block repositioning [14]. A second treatment option is an osteotomy with distraction [15]. Recently, a new technique, Orthodontic Bone Stretching (OBS), was proposed for ankylosed teeth repositioning with good results [9]. As this technique is successful for ankylosed teeth repositioning, it seems interesting to apply it for implant relocation. The aim of this paper is to present a case series describing the relocation of three maxillary incisors with a malpositioned implant, following the OBS procedure. The described cases were treated at Montpellier University Hospital.

## The concept of orthodontic bone stretching (OBS) to relocate dental implants

### Pre-surgical preparation

Bone level and implant osseointegration are controlled. The evaluation of the implant position includes pictures, dental casts, and imaging analysis. An orthodontic preparation is performed for malocclusion correction, particularly teeth misalignment, excluding the implant.

Before surgical treatment, special orthodontic preparation is necessary. On both sides of the implant, a space is opened to provide enough room for implant movement and repositioning. For bone vitality and the integrity of adjacent teeth, increasing the bone width of the septum is important to facilitate deep corticotomy in a safe way. Orthodontic anchorage is increased by a rigid archwire on the maxillary arch to avoid side effects during implant traction. At the end of orthodontic preparation, a bracket is bonded to the labial or palatal surface of the implant prosthodontic restoration, allowing immediate traction.

A dental computed tomography (CBCT) for surgical planning is then carried out before surgery to evaluate the distance between the implant and adjacent teeth, the bone density, and the cortical bone thickness, which are important factors for the OBS technique performing.

### Surgical technique

The patient medication comprises amoxicillin (1 g twice a day for 5 days), prednisolone (60 mg for 3 days), paracetamol (1 g three times a day for 2 days), and a 0.12% chlorhexidine solution (rinsing twice a day starting 24 h after surgery for 10 days).

**Fig. 2:**
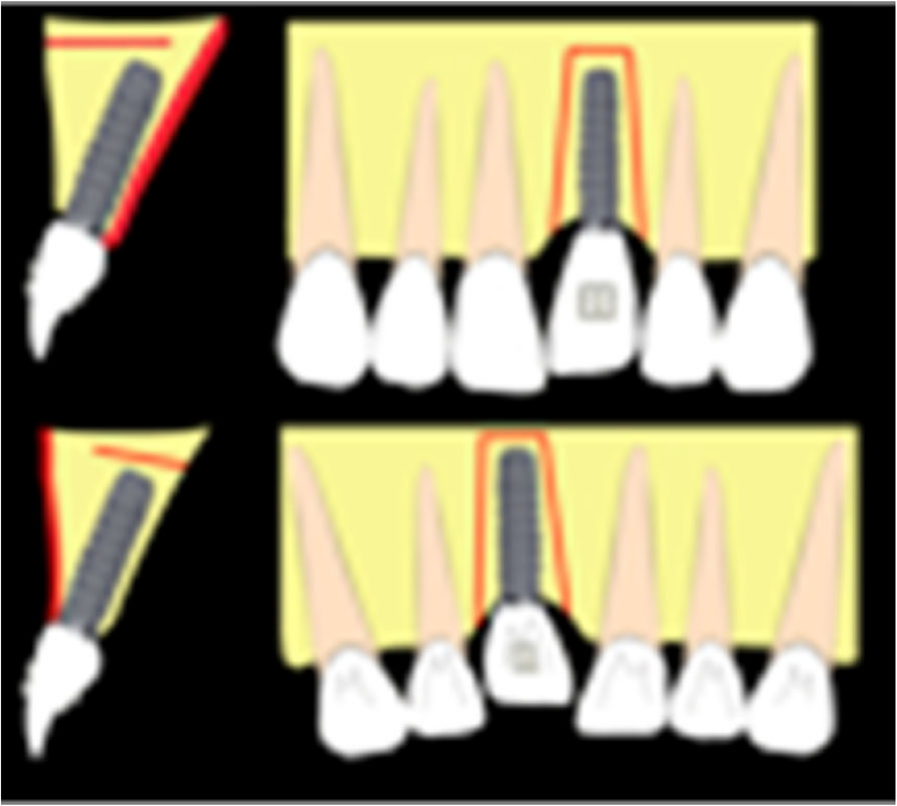
The deep corticotomies involve cortical bone and a large part of cancellous bone, preserving the opposite cortex, and can be done on the buccal or palatal side

Local anesthesia is done and, depending on the desired movement, surgery can be performed on the buccal side or the palatal side. Deep corticotomies involve the cortical bone and a large part of the cancellous bone but preserve the opposite cortex without any mobility of the implant area (Fig. 2).

For palatal side surgery, a palatal sulcular incision is made, including adjacent teeth to the implant, and a full-thickness mucoperiosteal flap is reflected for alveolar bone exposure. Surgical procedure is performed using a Satelec Piezotome2® with PZ1, PZ3, and BS1 or BS1L slim tips (Acteon Group®, Merignac, France). One vertical deep corticotomy cut is performed over the implant entire length on both sides, maintaining a safe distance from adjacent teeth, in the axis of the planned relocation. The two vertical incisions must be slightly convergent in the apical direction, to avoid movement blocking. Then, a third ultrasonic cut, positioned in the subapical apex area of the implant and connecting the two vertical cuts, is performed. For buccal side surgery, ultrasonic cuts are made on the buccal side. To preserve the implant soft tissue attachment and avoid recession, the incision for the full-thickness mucoperiosteal flap can be localized at the junction of the attached gingiva.

The mucoperiosteal flap is repositioned and sutured (Vicryl coated® No. 4-0 and 5-0; Ethicon, Issy-Les-Moulineaux, France).

### Postoperative management

Immediately after surgery, a continuous heavy orthodontic loading (from 150 to 200 g) is applied on the implant, along the same axis as the relocation. Orthodontic forces must not exceed the adjacent teeth anchorage value. Sutures are removed after 12 days. The patient is followed every 2 weeks by the orthodontist for traction reactivation until the relocation is ended. A complex implant- osseous movement is observed after one to 3 weeks. When the implant reaches the correct position, a new archwire is inserted, including the implant, for a period of three to 6 months to monitor the evolution. After removal of the appliance, if necessary, the prosthodontic restoration can be changed for a better esthetic result.

### CBCT measurement

Post-relocation CBCT is carried out. These data are compared with the pre-surgical CBCT by 3D superimposition using special software (CloudCompare®). Measurements are assessed in the three dimensions to control the implant movement and to evaluate the quantity of bone stretched during the OBS procedure.

## Case presentation

The OBS technique was approved by the ethical committee and the South of France Committee (Agreement CPP Méditerranée N°2014-A0006443). All patients provided written informed consent. The three cases were treated with the OBS technique for implant relocation.

### Case 1

**Fig. 3:**
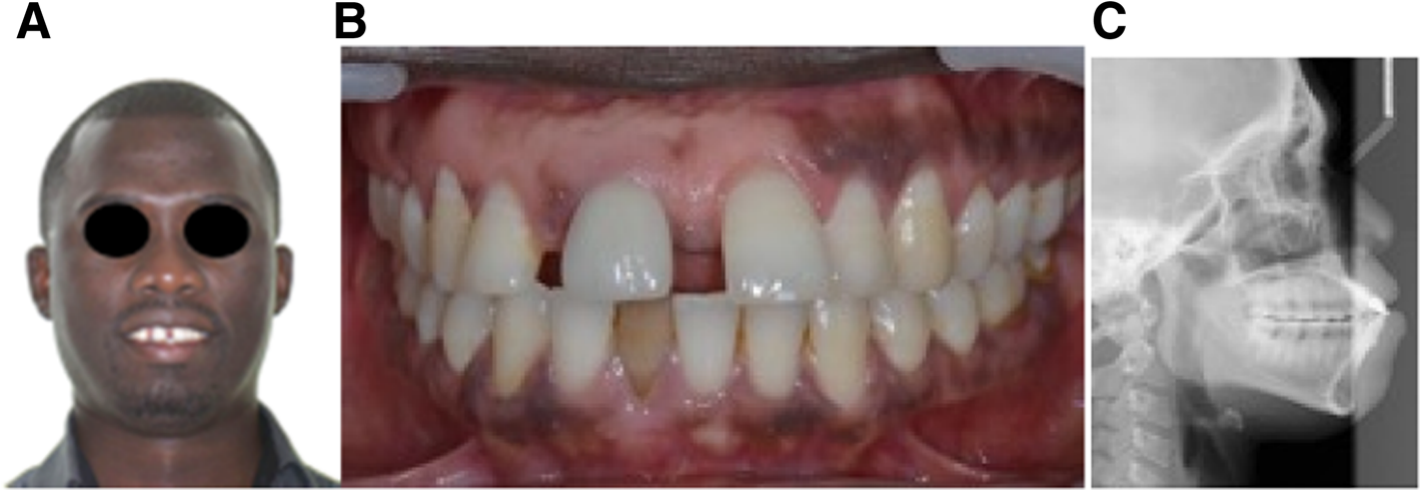
Initial situation in case 1. **A**, **B** Pre-treatment facial and intraoral photographs with the implant rehabilitation of maxillary right central incisor. **C** Pre-treatment lateral cephalogram

A 46-year-old man, in good general health, with an implant placed 5 years before, wanted to have his maxillary incisor diastema closed (Fig. 3). Clinical and radiological examination showed a correctly osseointegrated implant replacing the right maxillary central incisor and healthy soft tissues. The orthodontic treatment plan goal was to reduce the maxillary and mandibular incisor protrusion and the setup revealed, as the implant could not be moved by orthodontic treatment, a severe malposition without any possibility for a new prosthetic restoration. Esthetic rehabilitation required repositioning or implant removal.

The treatment management was, first, to correct the mandibular incisor protrusion with the avulsion of the right mandibular central incisor, which was necrotic with a very dark enamel. Then, the lower jaw space and the maxillary anterior diastema had to be closed, and finally, the implant had to be relocated or removed.

Orthodontic preparation to close the maxillary incisor diastema and the mandibular extraction space was done with a .018x.025 stainless steel wire (SSW) excluding the implant on the maxilla, and with a .019x.025 SSW in the lower arch.

**Fig. 4:**
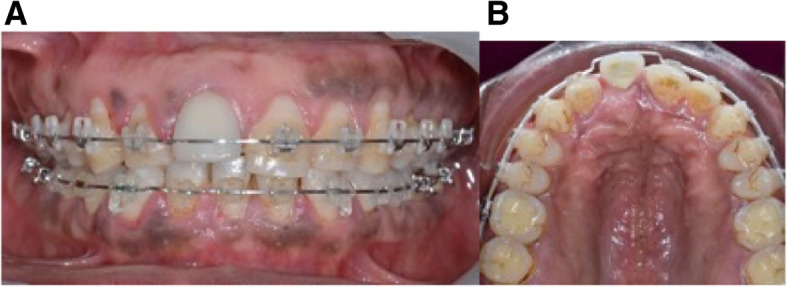
End of the orthodontic preparation excluding the implant in case 1. **A**, **B** Intraoral photographs, implant malposition is increased

**Fig. 5:**
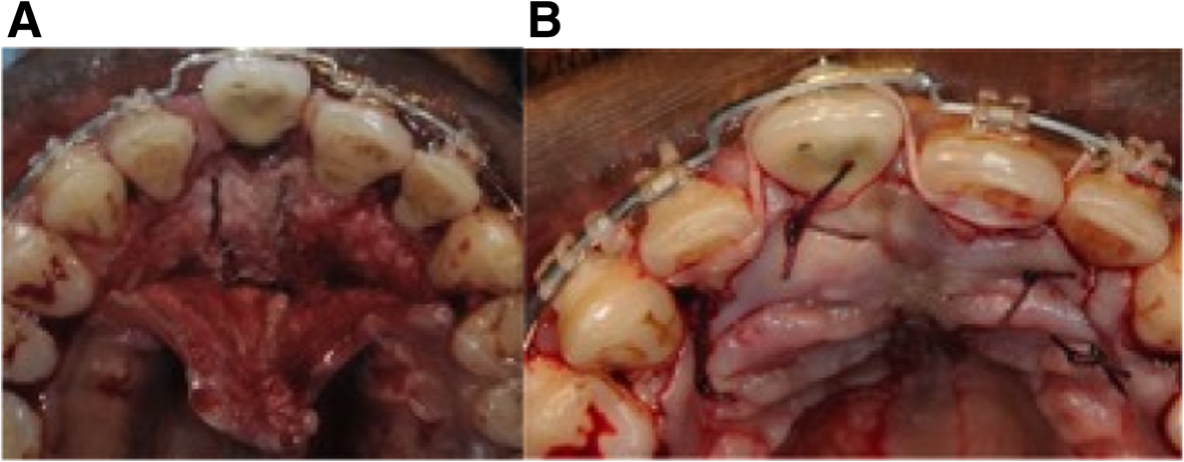
Surgery in case 1. **A** Deep corticotomies localized on the palatal side after flap reclinaison. **B** Suture and immediate heavy forces application by elastic chain

**Fig. 6:**
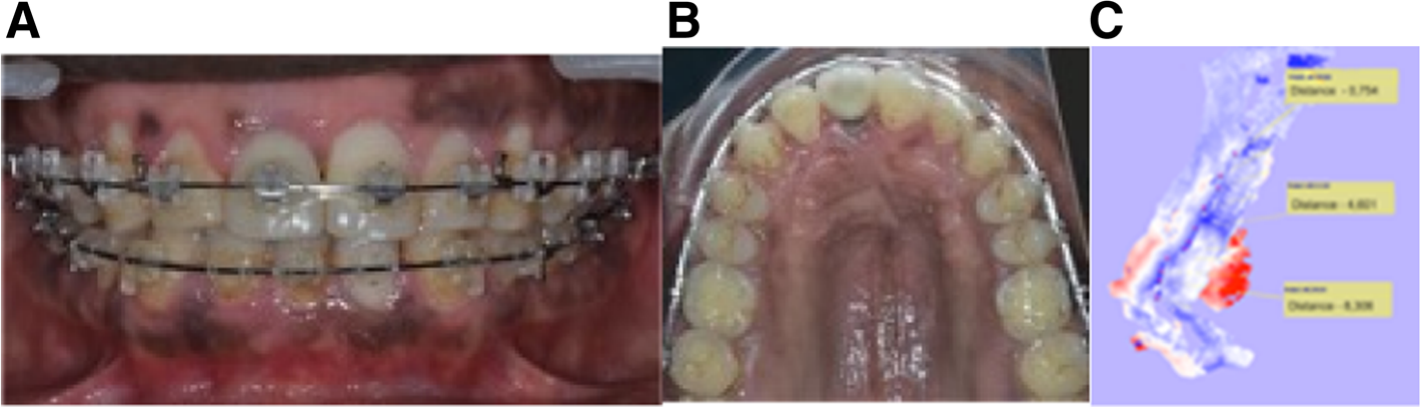
Situation before removal of appliance in case 1. **A**, **B**: Intraoral photographs after repositioning of 11 with OBS. **C** CBCT superimposition showing the implant movement in the palatal direction and the extrusion

**Fig. 7:**
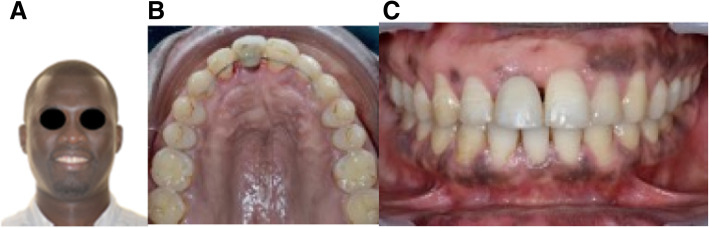
18 months post treatment case 1. **A**, **B**, **C** Facial and intraoral photographs, after periodontal maintenance therapy

After teeth alignment, it was confirmed that the position of the implant was unfavorable for a new prosthetic rehabilitation (Fig. 4). Despite good implant osseointegration and an adequate prosthetic crown, an OBS technique was proposed to the patient. Ultrasonic cuts were made on the palatal side for implant relocation with an antero-posterior movement. Immediately after flap closure, orthodontic forces were applied with an elastic chain between the implant and the archwire associated with 4.5 oz. intermaxillary elastics to pull the implant towards the palatal direction (Fig. 5). Elastics were changed by the patient twice a day, and, elastic chain reactivation was performed by the orthodontist, every week in the first month, then every 15 days. After 3 months, the implant crown was aligned with adjacent incisors, and the relocation result was stabilized by 3 additional months with a .021x.025 SSW. (Fig. 6). The prosthetic crown was preserved and a fixed retainer was bonded to the anterior upper arch. At 18 months post-treatment, teeth alignment and esthetic results were stable (Fig. 7).

### Case 2

A 23-year-old man, with an openbite facial pattern tendency, in good general health without any contraindication to surgery, was referred for prosthetic restoration of one implant replacing the central maxillary left incisor. The implant was placed 1 year after the extraction of the ankylosed tooth and was correctly osseointegrated, with healthy soft tissues.

**Fig. 8:**
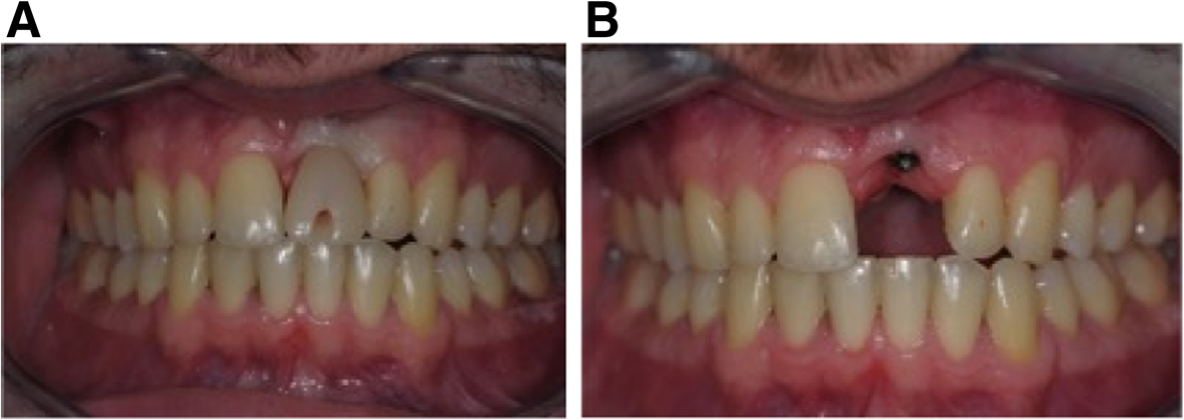
Initial situation in case 2. **A** Intraoral photograph showing the wrong position of the maxillary left central incisor implant. **B** Prosthetic space was too wide, implant position was too high with an inadequate angulation. Occlusion trouble was diagnosed on the left side

However, implant positioning did not respect gingival line alignment and worsened over time with an excessive labial angulation not permitting a screw-retained restoration. Orthodontic treatment and the OBS technique were indicated to realign the maxillary teeth and to relocate the implant in a good position, compatible with a screw-retained restoration, and to prevent a worsening implant position due to the continuous alveolar growth. A provisional screwed crown was made during the orthodontic preparation for esthetic considerations, but the restoration was too wide and the screw was positioned on the buccal side (Fig. 8).

**Fig. 9:**
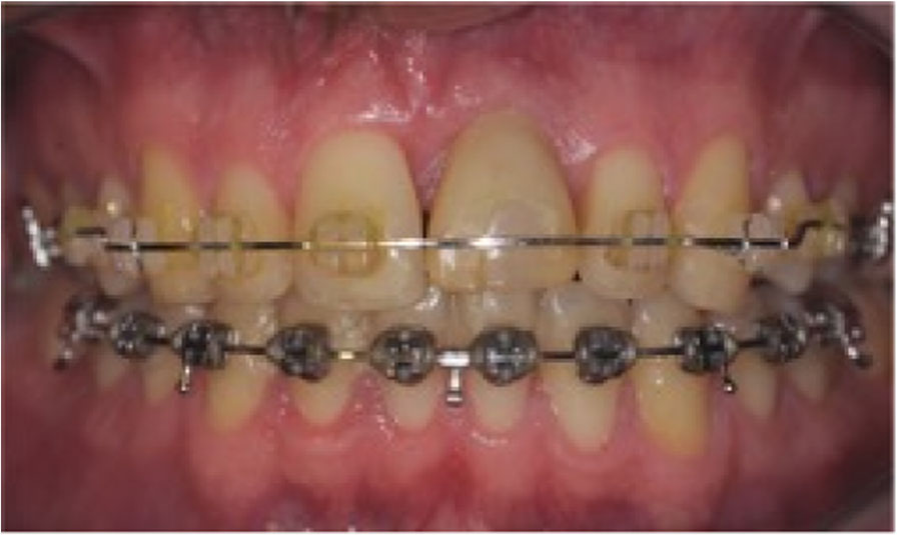
After orthodontic preparation in case 2. The prosthetic width of the provisional crown on the implant has been preserved to increase the space with the adjacent teeth

**Fig. 10:**
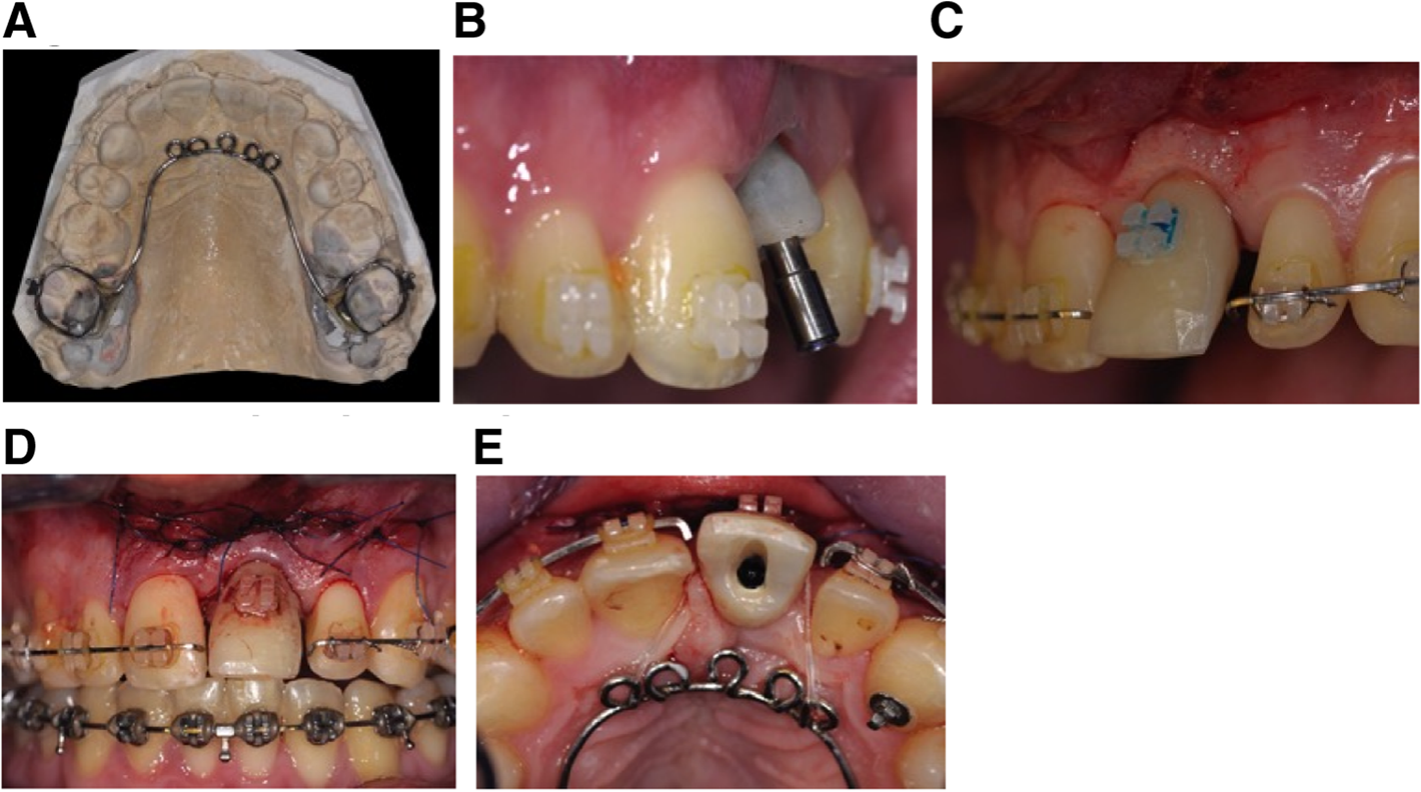
Pre-surgical and surgical treatment in case 2. **A** Trans-palatal device to transfer anchorage to the posterior teeth of the upper arch. **B** Impression of the position and the emergency profile of the implant. **C** The new temporary crown was in the right axis of the implant but not in an esthetic position. Space was opened. The view allows to evaluate implant relocation planned. **E**, **F** After surgery heavy force was immediately applied with elastic chain on the upper arch and intermaxillary elastic

The orthodontic treatment of the malocclusion was carried out and .021x.025 SSW were placed on both arches with additional hooks on the lower arch (Fig. 9). At the end of the preparation, a new provisional restoration with an anatomical shape and dimension was built in the implant axis according to the final realization. The maxillary archwire was adapted, allowing the insertion of the new provisional screwed crown in the implant axis to evaluate the real wrong position of the implant and for movement guidance during the orthodontic implant traction. (Fig. 10).

**Fig. 11:**
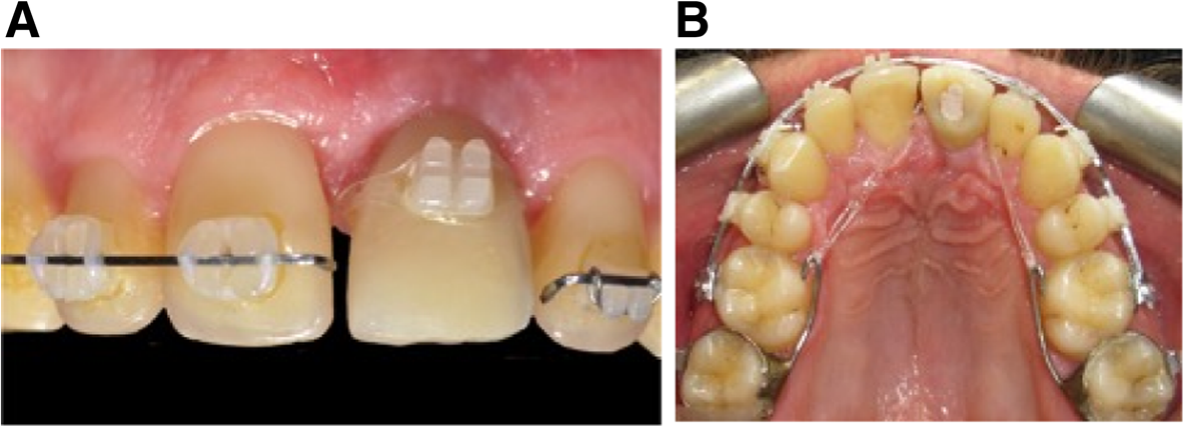
Relocation of the implant case2. **A**, **B** At the end of the repositioning, the spaces may be closed. Trans-palatal device has been modified to avoid blocking of the palatal movement of the temporary screwed crown

During surgery, only one horizontal incision was made in the keratinized gingiva, allowing papillae preservation and a full-thickness mucoperiosteal flap elevation on the buccal site. Two vertical deep ultrasonic corticotomies, on either side of the implant, were performed, rising in the direction of the incisal edge under the soft tissues that remained attached. One horizontal osteotomy was added apically connecting the two vertical osteotomies. A trans-palatal device was placed for anchorage to control the anterior teeth position. Immediately after surgery, continuous orthodontic traction was placed using an elastic chain connecting the trans-palatal device and the implant, plus an inter-maxillary elastic from the bonded bracket on the implant crown and the lower archwire hooks. To facilitate implant movement during traction, the trans-palatal arch was modified. After 3 months of traction, the implant with the temporary crown was in a good position, and the remaining space was closed. Stabilization was done with a final .021x.025 SSW for a period of 3 months. A new screwed zirconium rehabilitation was made and, after orthodontic device removal, a retainer was bonded, including the final prosthetic restoration. (Fig. 11).

### Case 3

**Fig. 12:**
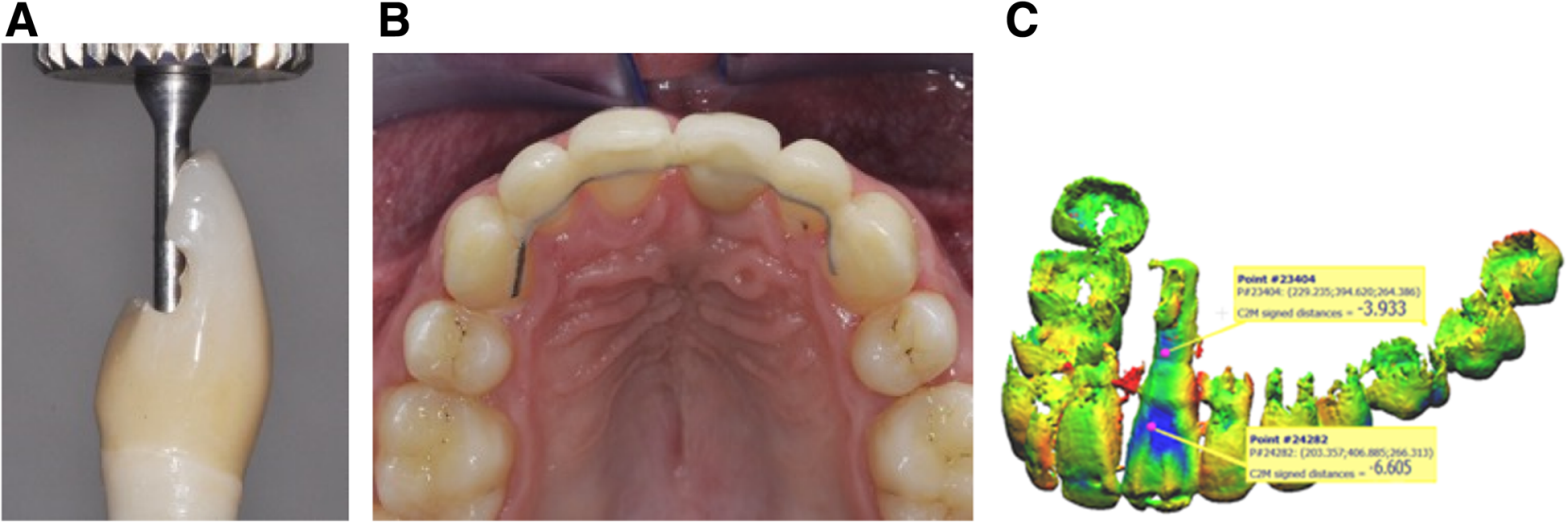
ABC post-treatment in case 2. **A**, **B** A new prosthetic rehabilitation was realized and screwed into the axis of the implant. A space has been created in the zirconia crown to receive the bonded retainer including the prosthetic restoration. **C** CBCT superimposition showing the implant movement in the palatal direction

A 64-year-old woman was referred to correct the prosthodontic restoration on the right maxillary first incisor with infraocclusion. This patient had been treated by implant therapy 20 years before. She was in good general health and did not present any contraindication to surgery procedure, but she had a high tobacco consumption, which she had decided to reduce. Clinical examination showed chronic periodontitis, the replacement of the right maxillary first incisor by an implant with infraocclusion, and a crown with a slight palatal position (Fig. 12). Soft and hard tissues around the implant and osseointegration were stable. The facial skeletal pattern was normodivergent. The actual implant position was due to 20 years of continuous alveolar growth. Considering the good palatal implant positioning at the time of the surgery, 20 years before, the right axis was kept, without any buccal side effect movement. A new screwed restoration would have been possible, but the prosthetic rehabilitation to compensate for the infraocclusion would have led to a disappointing result, with too long a crown and an incorrect gingival line alignment. For this reason, periodontal treatment was planned first before orthodontic preparation and implant relocation at the right level using the OBS technique.

**Fig. 13:**
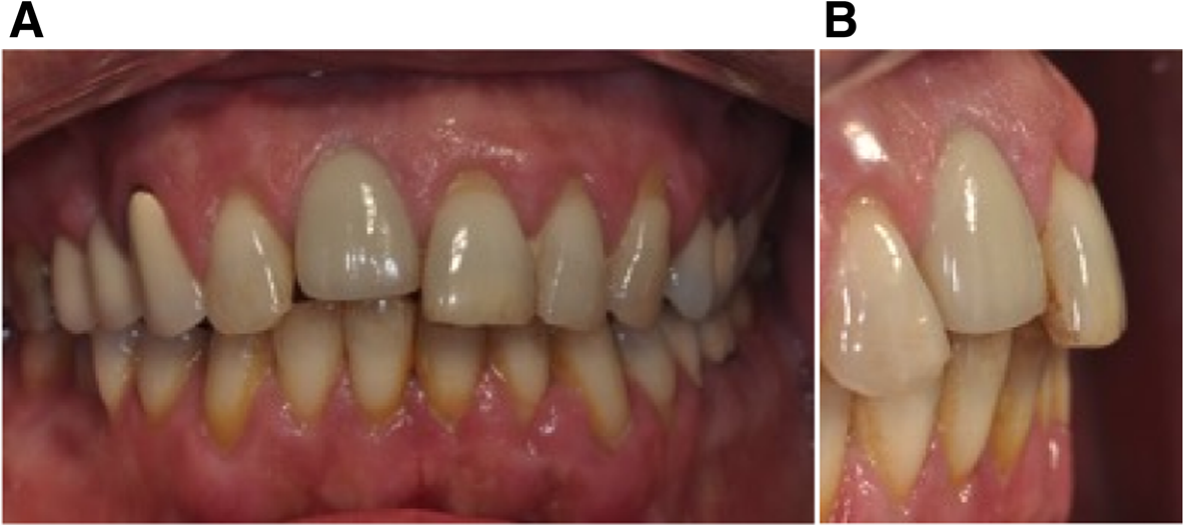
Initial situation in case 3. **A**, **B** Infraocclusion of the right central incisor maxillary single-tooth implant with a slightly tilted axis of the implant towards the palate, compared to the adjacent teeth

Orthodontic preparation consisted of teeth alignment excluding implant and a .021x.025 SSW was placed on the maxillary and mandibular arches before surgery. When the alignment phase was completed, infraocclusion on the incisor prosthodontic crown was severely worsened, compared to the incisal edge of the adjacent teeth (Fig. 13A). Immediately before surgery, an overlay .016 Nickel-Titanium archwire was placed on maxillary teeth, including the implant. (Fig. 13).

**Fig. 14:**
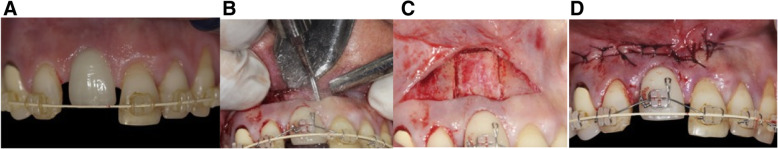
**A** After orthodontic preparation, the spaces between the adjacent teeth were opened and the real infraocclusion was evaluated. **B**, **C** Surgical procedure is performed using a Satelec Piezotome2® with PZ1 and slim tips (Acteon Group, Merignac, France). **D** After suture an immediate and continuous orthodontic traction was applied with a nickel-titanium archwire (including the implant) and with intermaxillary elastic

**Fig. 15:**
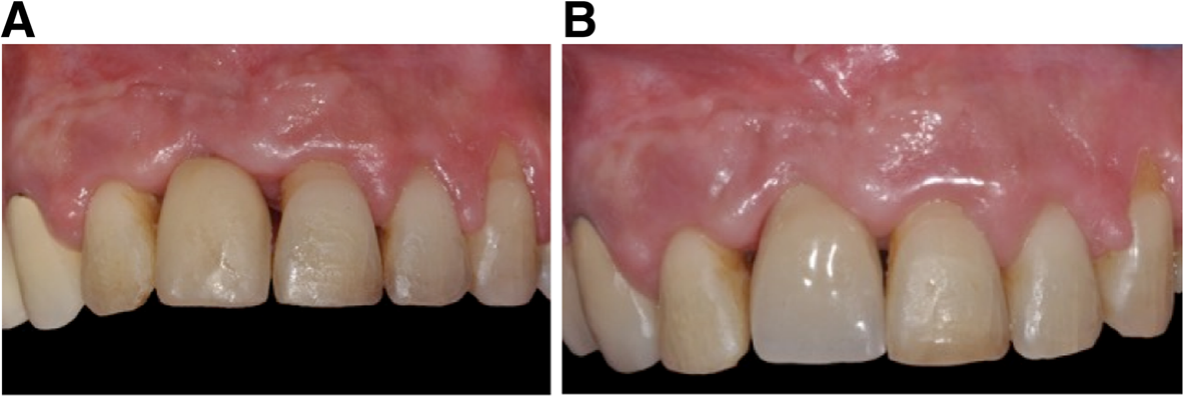
**A**, **B** post treatment in case 3. **A** One month after treatment, a retainer was bonded including the implant. **B** two years post treatment, plaque control was not ideal, but a periodontal stabilization occurred

The OBS procedure was performed on the buccal side with preservation of the implant soft tissue attachment. Immediately after surgery, orthodontic forces were applied. Every 2 weeks (Fig. 14), the patient was followed by the orthodontist for traction reactivation. After 2 months of orthodontic traction, the movement was stopped. New surgery was planned on the palatal side. Immediately after surgery, orthodontic traction was applied along the desired axis. Bone stretching movement allowed implant extrusion. After stabilization, a new crown was made and a retainer was bonded (Fig. 15).

## Results

No problems were encountered during surgeries and the orthodontic traction period. In these three cases, control CBCTs showed no problem with implant repositioning or osseointegration. Superimpositions between pre and post-surgery images demonstrated the movement due to bone stretching phenomena.

In case 1, after implant repositioning, it was not necessary to have a new prosthodontic restoration. Orthodontic treatment and OBS modified facial and smile esthetics. The implant movement in the palatal direction was 0.56 mm at the apex and 6.56 mm at the incisal edge (Fig. 6c).

In case 2, after 4 months of traction, the implant was correctly repositioned, and final prosthodontic restoration was done. CBCT superimpositions showed a movement at the implant-abutment junction of 3.93 mm in the palatal direction and 6.60 mm at the incisal edge. (Fig. 12c).

In case 3, the movement in the occlusal direction was difficult but the implant was repositioned after 6 months (4 months after the second surgery). Displacement was measured at 3.88 mm at the incisal edge and new prosthodontic restoration was done to obtain a better esthetic result. Preventive periodontal maintenance was carried out, but plaque control was very poor.

## Discussion

Improperly positioned implants can be caused by problems during surgery, such as the impossibility of placing the implant in an adequate position or an initial positioning error. The need for orthodontic treatment for a patient with an implant in the anterior sector is also problematic as all the teeth could be moved except the implant, which cannot be relocated with an orthodontic appliance only. Infraposition or buccal position can also occur as the result of a lack of alveolar growth around the osseointegrated implant in comparison with adjacent teeth as they continue their vertical alveolar growth. For implant relocation, segmental osteotomies were proposed with good results, as shown in case reports. In this procedure, the bony-implant block is repositioned and immediately stabilized in the new position by a rigid fixation ( [14, 16–19]). The bone graft can be associated with bone-block movement [20]. In a retrospective study including 15 cases, Stacchi et al. [21] showed that implant repositioning with osteotomy to correct unrestorable malpositioned implants could be stable. The alveolar segment vascularization is essential for this technique and depends on soft tissue attachment for bone preservation. Soft-tissue stretching can be a limiting factor for vascularization and implant-osseous complex movement. Moreover, it is very important to prevent micro-movements of the mobilized block that could disrupt the healing process [22]. Implant positioning must be immediately perfect due to the rigid and unmodifiable fixation after surgery.

The distraction osteogenesis procedure was proposed to reduce these challenges [14, 23]. After osteotomies and callus formation, heavy strength induces a gradual movement of the mobilized block, with a progressive augmentation of soft and hard tissues. The main difficulty of this process is to manage the block in all directions of the space. The distractor allows movement only in its axis direction but not in all other directions, which can lead to incorrect distraction vectors. Special devices were proposed to improve movement with good results [24], but this technique remains complex and increases cost.

The OBS technique was developed for ankylosed teeth relocation [9], and its application seems interesting for implant repositioning. In OBS, the osteotomy is limited to the buccal (or palatal) side of the alveolar bone. Vascularization is preserved by keeping a portion of the bone and the attached soft tissues. A block stabilization is not necessary as partial osteotomies leave the implant-bone complex completely immovable. These deep corticotomies are less traumatic than complete osteotomies and facilitate implant movement by decreasing resistance of the partial bone-implant block. Before surgery, the orthodontic force cannot move the implant because the dental arch anchorage is weaker than the resistance of the osseointegrated implant. After OBS surgery, implant relocation without ligament is allowed by bone movement. In all cases, as with ankylosed tooth relocation, the displacement begins after two or 3 weeks of traction, The OBS technique appears to be a bone stretching phenomenon using the implant as anchorage. Our experience showed that orthodontic preparation is very important and is associated with good management of the tissues around the implant. It requires enough distance from adjacent teeth to allow deep corticotomy cuts while leaving enough bone on each side to preserve the vascularization of the septum for implant relocation, at least 3 mm.

Vertical cuts must be slightly convergent in the apical direction and in the same axis as the orthodontic traction. If the loading force has a different axis or if corticotomies converge in the occlusal direction, the implant will not move. Moreover, the applied orthodontic force needs to be immediate, continuous, and heavy, preventing healing in bone cuts areas and stretching the residual palatal or buccal bone.

Furthermore, animal studies have shown that corticotomy increases the turnover of bone [25] and particularly of alveolar bone [26]. For the OBS procedure, the use of piezo cuts is preferred to any other technique because of its precision and the slim profile of the ultrasonic saw. Moreover, surgery with piezotome, compared with conventional instrumentation leads to significantly higher bone activity [27]. It may be possible that the biological effect of ultrasonic cuts facilitates movement during OBS. In one case (case number 3), a second surgery was necessary, because movement stopped. As this patient was a heavy smoker, nicotine could have modified the quality of the OBS movement. In orthopedic surgery or bony distraction, nicotine decreases bone turnover and increases surgical complications [28, 29]. The consequences of smoking during orthodontic treatment have been described with an increase in treatment time and a decrease in tooth movement [30].

The OBS technique is different from bone distraction because the applied forces are immediate and continuous. Bone cuts are not complete, and waiting for a callus is not indicated to stretch residual bone. The applied forces are lighter (150–200 g), and the orthodontic appliance manages the movement in all directions, unlike distraction, where the force vector is unidirectional and unmodifiable during treatment.

This case series showed the conservation of the implant osseointegration despite bone- implant complex movement. Osseointegrated implants used as orthodontic anchorage showed a force resistance, without peri-implant bone changes in animal models or human use [31, 32]. Melsen & Lang, in an animal study, have applied orthodontic forces of 100, 200, and 300 g in three different osseointegrated implant groups. Osseointegration degree expressed by direct bone-to-implant contact (B.I.C) was not influenced by loading force, and for 11 weeks of applied forces, no clinical implant mobility or peri-implant probing depth exceeding 3 mm could be observed [33]. If immediate orthodontic forces, from 200 g to 600 g, are applied on the implant, they do not interfere with the osseointegration phenomenon, and they do not decrease the amount of B.I.C observed [34, 35]. During the osseointegration period, controlled and progressive continuous orthodontic force from 100 to 300 g (with an increase of 100 g every 3 weeks) increases the bone-implant contact in an animal model [36].. On the other hand, a recent study by Becker et al. [37] showed, in an animal model, that implants can migrate through the bone under applied forces during osseointegration. In the OBS technique, after osseointegration, the implant movement under heavy orthodontic forces is a bone movement with the stretching of the residual preserved cortical bone.

## Conclusion

The OBS technique seems interesting as it provides surgeons with another option for the correction of implants in an incorrect position. The possibility to manage movements in all directions allow driving the implant towards the chosen final position. This technique can be combined with orthodontic treatment, which is often necessary for the esthetic rehabilitation of anterior implants in the wrong position. Further controlled studies would be highly beneficial to determine the predictability of the OBS procedure and compare it with osteotomy and distraction.

## Data Availability

Due to the nature of this research (case series), participants of this study did not agree for their data to be shared publicly, so supporting data is not available.
